# Conflict and COVID-19 in Yemen: beyond the humanitarian crisis

**DOI:** 10.1186/s12992-021-00732-1

**Published:** 2021-07-22

**Authors:** Mohammed Alsabri, Ayman Alhadheri, Luai M. Alsakkaf, Jennifer Cole

**Affiliations:** 1 Pediatrics, 1 Brookdale University Hospital and Medical center 1Brookdale Plaza, Brooklyn, NY 11212 USA; 2 Emergency Department, Al Thawra Modern General Hospital (TMGH), Sana’a City, Yemen; 3 Emergency Medicine, McLaren Oakland Hospital, 50 N. Perry St, Pontiac, MI 48342 USA; 4 Department of Geography, Royal Holloway University of London. Egham Hill, Egham, Surrey, TW20 0EX UK

**Keywords:** Yemen, COVID-19, War, Humanitarian crisis, Disaster

## Abstract

**Background:**

Yemen has been left in shambles and almost destroyed by its devastating civil war, and is now having to deal with the spread of coronavirus. The Yemeni people have been left to fend for themselves and faced many problems such as hunger, the ongoing war, infections, diseases and lack of equipment even before the COVID-19 pandemic. All together it is a humanitarian crisis. Only around 50% of the hospitals and healthcare facilities are in full working condition, and even those that are functioning are operating at nowhere near full potential. Healthcare staff and facilities lack necessary essential equipment and money.

**Conclusion:**

As, sadly, is common in conflict-affected regions, the violence has brought with it a secondary disaster of infectious disease outbreaks. Yemen is not only battling COVID-19 amid a catastrophic war, but also has to deal with other diseases such as cholera, diphtheria and measles. A number of key measures are needed to support the current efforts against this deadly epidemic and its potential subsequent waves as well as to prevent further epidemics in Yemen.

## Background

Yemen is a country in crisis. Located at the southern tip of the Arab Peninsula it was once a beautiful country but in recent decades has been devastated by civil war. In September 2014, the capital city of Sana’a was taken by armed insurgents; in March 2015 a Saudi Arabian coalition launched airstrikes in retaliation, escalating matters into a full-blown conflict that is still ongoing. The aftermath, which has left the Yemeni people facing hunger, disease and a lack of medical equipment, is considered to be the world’s worst humanitarian crisis [1] . In addition, Yemen is frequently impacted by natural disasters including drought, heavy rains, and floods. Recently, the COVID-19 pandemic has added to the challenge. Today, only around 50% of Yemeni hospitals and medical facilities are in full working condition and even these are in dire need of essential equipment and proper funding [2]. The war has caused an estimated 233,000 deaths and 24.3 million Yemenis – more than 80% of the population – currently require some form of humanitarian assistance [1]. More than 70% lack safe drinking water or adequate sanitation. Infectious disease outbreaks are, unsurprisingly common: 205,662 suspected cholera cases were recorded from 1 January to 30 October 2020; 5801 probable cases of diphtheria between August 2017 and September 2020; 6777 suspected cases of dengue in 2020 and 1744 cases of measles (nearly double the amount in the next highest country) [3, 4]. Looking even further back, since 2017 there have been over 2 million cases of cholera, described by both UNICEF and WHO agencies as the worst documented epidemic in the history of cholera [5] . Dengue fever and other infections, such as chikungunya, have proven to be hurdles and often lead to initial misdiagnoses of COVID-19 as they have extremely similar symptoms and presentations [4, 6, 7]. According to the WHO, since 3 January 2020, Yemen has had 6812 confirmed cases of COVID-19 with 1336 deaths [6]. Regarding COVID-19, national interventions seem to be delayed or sometimes neglected. It is not clear whether the biggest challenge comes from lack of funding, lack of knowledge, other causes, or the combination of multiple causes. We have not seen any well-structured epidemiological studies that help in understanding the origin and distribution of cases like those seen in other nearby countries such as Oman [8]. Global warming is another potential crisis looming on the horizon. The COVID-19 pandemic has shown what a modern global crisis looks like. Conclusions must be drawn [9] and applied to other global crises [10, 11]. Klenert et al. identified five lessons from the COVID-19 crisis that must be applied to address global warming including: urgent need to start mitigation, the cost of delayed intervention, and the need for global intervention [10]. Global problems must not be sidelined. Studies continue to quantify the challenge and emphasize its risks, which may potentially lead to an even greater crisis than COVID-19 [11] but countries such as Yemen do not have the resources to cope, as this crisis has shown.

### Disaster preparedness/emergency medicine: current status in Yemen

Sadly, the unstable political atmosphere in Yemen has given little chance for improvement in the field of disaster preparedness [12]. A recent study from 2018 surveyed knowledge and attitudes of 531 Yemeni health professionals [13]. Only one third of health professionals have good knowledge of disaster management and 40% have not been taught disaster preparedness at all. As of now, there is no formal prehospital Emergency Medical Services (EMS) in Yemen nor a local equivalent of a 911 or 999 number to call in case of a medical emergency. In mass casualty incidents – which are very common – it is not unusual for Yemeni ER (Emergency Room) physicians to receive victims who have been transported in private cars by people who were present at the scene [13]. To the best of our knowledge, across the country, there is no formal training pathway approved or available for paramedics. But there is hope. An emergency medicine program was implemented in Yemen in 2004. In December 2013, less than a week after a terrorist attack on the Defense Medical Complex Hospital in Sana’a, a group of physicians established the Yemeni Association of Emergency Medicine and Disasters (YAEMD). This organization has been a full member of the International Federation for Emergency Management (IFEM) since January 2014 and held its first annual international in Sana’a on 28-29th January 2019. In 2020, the Yemeni Board of Emergency Medicine (YBEM) was established. At the time of writing, there are two training centers and YBEM is working on opening at least one more. All three are situated in Sana’a, however, leaving them extremely vulnerable to the ongoing violence. Many of Yemen’s home-trained EM specialists have migrated outside the country, looking for better salaries and safer conditions elsewhere.

### The current status and future of emergency medicine in Yemen

Airstrikes, ground attacks, military occupation and assaults on health workers are all common occurrences in Yemen. Medical facilities are not immune to terrorist attacks and the staff who work in them are poorly equipped and underpaid: it is hardly surprising many migrate to escape such a challenging working environment. As efforts are made to improve Yemen’s healthcare system, emergency care must be part of such discussions. The nation needs to work at identifying gaps and flaws in its healthcare systems. Improving research activities is needed in order to establish basic data that will help to improve health service delivery. There must be a more focused approach to preventive medicine, for example, and a better understanding of chronic disease within the local population to help to ensure scarce resources are allocated most effectively. The problems with Yemen’s emergency medical services are systematic: the healthcare system is under-funded and under-resourced overall. In a country with scarce budget available, few resources, and poorly compensated emergency medicine staff and physicians, a depleted workforce due to mass outward migration of qualified professionals is somewhat inevitable. Systematic revisions to the health care system, rebuilding PHC (primary health care) facilities, guaranteed universal insurance plans, a greater emphasis toward preventive medicine and continued training in emergency medicine, are needed to help overcome these challenges.

## Conclusion

This paper presents the accounts of Yemeni emergency physicians who comment on the distinctive challenges that face Yemen’s emergency departments. These challenges include epidemic diseases such cholera, diphtheria, dengue and measles; the geopolitical impact of the ongoing civil war; and daily threats of violence and homicide against emergency healthcare workers. Potential solutions include national and international efforts to properly allocate government funds and to implement universal health insurance plans. Future works and resources should be allocated to addressing these challenges in both short term reactionary measures as well as longer term preventative measures. The solutions to Yemen’s current situation need to be multifaceted, with approaches that target the challenges from multiple angles. Solutions must come from educational and training programs dedicated to filling in the gaps throughout the short and longer-term interventions. Solutions might include community and neighborhood involvement, and invoking assistance from volunteers and activists of all ages to pilot smaller sized improvement projects. Lastly, a systems approach to emergency and disaster management is needed.

## Data Availability

Not applicable.

## Abbreviations

EMS: Emergency Medical Services
ER: Emergency Room
PHC: Primary health care
YAEMD: Yemeni Association of Emergency Medicine and Disasters
IFEM: International Federation for Emergency Management
YBEM: Yemeni Board of Emergency Medicine

## References

[1] Global Humanitarian Overview 2021. Geneva: United Nations Office for the Coordination of Humanitarian Affairs, 2020. https://www.unocha.org/global-humanitarian-overview-2021.

[2] Al-Awlaqi S, Dureab F, Annuzaili D, Al-Dheeb N (2020). COVID-19 in conflict: the devastating impact of withdrawing humanitarian support on universal health coverage in Yemen. Public Health Pract.

[3] Global measles outbreaks, 2020. URL: https://www.cdc.gov/globalhealth/measles/data/global-measles-outbreaks.html. Accessed 1 April 2021.

[4] Alsabri M, Nightingale B, Amin M, Cole J (2020). When COVID-19 hit Yemen: dealing with the pandemic in a country under pressure from the world’s worst humanitarian crisis. Global J Med Public Health.

[5] Statement from UNICEF Executive Director Anthony Lake and WHO Director-General Margaret Chan on the cholera outbreak in Yemen as suspected cases exceed 200,000 [Internet]. UNICEF. 2020 [cited 2021Jan16]. Available from: https://www.unicef.org/press-releases/statement-unicef-executive-director-anthony-lake-and-who-director-general-margaret. Accessed 1 April 2021.

[6] Yemen: WHO Coronavirus Disease (COVID-19) Dashboard With Vaccination Data [Internet]. World Health Organization. World Health Organization; [cited 2021Jun6]. Available from: https://covid19.who.int/region/emro/country/ye. Accessed 1 April 2021.

[7] A Tipping Point for Yemen’s Health System: The Impact of COVID-19 in a Fragile State [Internet]. MedGlobal. 2020 [cited 2021Jun6]. Available from: https://medglobal.org/yemen-covid-report-july2020. Accessed 1 April 2021.

[8] Al-Kindi KM, Alkharusi A, Alshukaili D, Nasiri NA, Al-Awadhi T, Charabi Y (2020). Spatiotemporal Assessment of COVID-19 Spread over Oman Using GIS Techniques.

[9] Cole J, Dodds K. Unhealthy geopolitics: can the response to COVID-19 reform climate change policy? Bull World Health Organ. 2021;99(2):148-54. 10.2471/BLT.20.269068. 10.2471/BLT.20.269068 PMC7856368 33551508

[10] Klenert D, Funke F, Mattauch L, O’Callaghan B. Five Lessons from COVID-19 for Advancing Climate Change Mitigation: Environmental and Resource Economics. Springer Netherlands; 2020. [cited 2021Jun6]. Available from: https://link.springer.com/article/10.1007%2Fs10640-020-00453-w 10.1007/s10640-020-00453-w PMC7397958 32836842

[11] Valipour M, Bateni SM, Jun C (2021). Global surface temperature: a new insight.

[12] Aladhrai SA, Djalali A, Corte FD, Alsabri M, El-Bakri NK, Ingrassia PL (2015). Impact of the 2011 revolution on hospital disaster preparedness in Yemen. Disast Med Public Health Prepared.

[13] Naser WN, Saleem HB (2018). Emergency and disaster management training; knowledge and attitude of Yemeni health professionals-a cross-sectional study. BMC Emerg Med.

